# Systematic Development of Intelligent Systems for Public Road Transport

**DOI:** 10.3390/s16071104

**Published:** 2016-07-16

**Authors:** Carmelo R. García, Alexis Quesada-Arencibia, Teresa Cristóbal, Gabino Padrón, Francisco Alayón

**Affiliations:** Institute for Cybernetics, Campus de Tafira, Las Palmas de Gran Canaria, University of Las Palmas de Gran Canaria, Las Palmas 35017, Spain; teresa.cristobalb@gmail.com (T.C.); gabino.padron@ulpgc.es (G.P.); francisco.alayon@ulpgc.es (F.A.)

**Keywords:** ubiquitous computing, intelligent transport systems, service-oriented architectures, public transport management

## Abstract

This paper presents an architecture model for the development of intelligent systems for public passenger transport by road. The main objective of our proposal is to provide a framework for the systematic development and deployment of telematics systems to improve various aspects of this type of transport, such as efficiency, accessibility and safety. The architecture model presented herein is based on international standards on intelligent transport system architectures, ubiquitous computing and service-oriented architecture for distributed systems. To illustrate the utility of the model, we also present a use case of a monitoring system for stops on a public passenger road transport network.

## 1. Introduction

Transport systems are a feature of many people’s lives and therefore have a material impact on their quality of life. It is estimated that 40% of the world population spend at least one hour of their time on the road every day [1], and people are becoming more and more dependent on transport systems. Because of this dependency, which involves large-scale use of the various modes of transport, transport systems must meet a number of challenges that are of vital importance to modern societies. The first of these challenges is the problem of congestion in many parts of the world, mainly in the most populated urban areas. Congestion leads to increased fuel consumption, pollution levels and difficulties in deploying mobility plans based on the use of public transport [2]. According to medical studies, all of these factors result in a deterioration of public health because they increase the risk of heart and respiratory diseases [3]. According to the World Health Organization, the number of deaths per year due to health problems caused by pollution is around seven million people. The second challenge posed by the growing use of transport systems is the increased risk of accidents, particularly in developing countries. In the European Union, every year 250,000 people are seriously injured; 28,000 people died in 2012 and 25,700 in 2014 [4]. Various researchers and agencies have concluded that most accidents result from human error; indeed, Malta [5] concluded that three quarters of accidents are due to human error. We should also point out that accidents are a major cause of delays due to congestion problems. The United States Department of Transportation (DOT) issued a report in 2000 in which it estimated that between 50% and 60% of delays were due to traffic accidents [6]. Therefore, there is a clear need to reduce both the number of accidents and their impact. Finally, there are more and more physical, land-use restrictions in the building of new infrastructure, such as roads and highways. After the September 11 terrorist attacks in New York in 2001, it became clear that another aspect had to be considered when evaluating the effectiveness of a transport system: the capacity to manage emergency situations, for example, the ability to deal with mass evacuations using the transport network. Ultimately, the competitiveness of a country, its economic strength and even its security rely heavily on its transport systems [7].

Some of the challenges identified in the previous paragraph may be met by applying specific transport regulations. For example, during the Olympic Games in Beijing, restrictions were introduced on the use of vehicles on alternate days depending on whether their license plate ended in an odd or an even number. This rationing strategy reduced private traffic levels in the city by 50% during this event. Such measures considerably reduce congestion and pollution problems, but are only effective in exceptional situations—when major events take place—because in everyday situations they have proven to be ineffective. Another strategy is to increase the capacity for vehicle movement by building new road infrastructures. However, land is a scarce and therefore expensive resource, not to mention the maintenance costs of these infrastructures. Finally, another field of action is the use of Information and Communications Technology (ICT) in improving transport systems. The study presented in this article pertains to this line of action.

Like other industrial sectors, the transport sector has benefited from advances in communications, especially mobile communication technologies, computer systems and sensor technologies. Technological advances in the transport sector since the 1970s have given rise to a highly significant field of research called Intelligent Transport Systems (ITS). This paper aims to contribute to this field by proposing an architecture model for the systematic development of advanced telematics systems to improve public passenger transport by road. These systems are deployed and executed intelligently on the transport network, making it more efficient, safe, accessible and attractive to the user. For the systems to have a high level of integration, interoperability and flexibility, the model has used the principles and recommendations of ITS architecture standards and the service-oriented architecture model for distributed systems (SOA). For the systems to run intelligently, and to autonomously process data provided by the various infrastructure elements, the model was based on the principles of ubiquitous computing, in particular, context awareness.

This article is structured as follows: the following section describes the objectives and challenges to be met by the model; the third section describes related studies; the fourth section describes the architecture model; in the fifth section a use case of this model is described, in which a system for monitoring bus stops is developed; and finally, the main conclusions and future lines of work are presented in the sixth section.

## 2. Objectives and Challenges

The field of ITS comprises different system types that can be classified as follows: Road and Highway Management Systems, Freight Management Systems, Incident and Emergency Management Systems, Transport Management Systems, Traveler Information Systems and Public Transport Information Management Systems. The aim of our proposed architecture model is to provide a framework in which to systematically develop and deploy intelligent systems pertaining to the latter three types on this list. The general objectives of each of these types of system are as follows:

- Transport Management Systems: The aim of these systems is to improve the efficiency, reliability and safety of transport system operations.
- Traveler Information Systems: The aim of these systems is to provide traveler information irrespective of type (private or public) and mode of transport used (sea, air or land). The scope may be regional, national or even international.
- Public Transport Information Management Systems: These systems are based on the use of all types of data related to transport systems and their purpose is to generate knowledge to help understand past and present and predict the future of transport systems.

**Figure 1:**
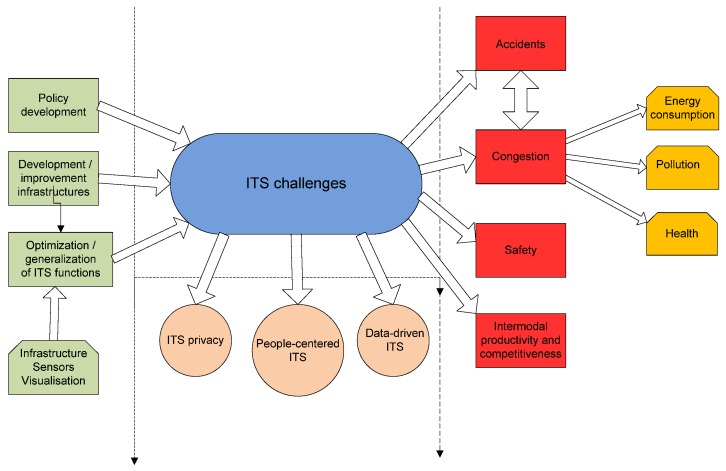
Overview of Intelligent Transport Systems (ITS): activities, challenges and problems.

Figure 1 offers a comprehensive overview of ITS. On the left are the activities carried out by authorities and bodies responsible for transport systems; in the center, the main challenges or objectives of ITS and, on the right, the problems to be solved and consequences thereof. Management of ITS-based transport systems requires continuous monitoring of what is happening in the transport systems and feedback for system improvement and development. The focus is always on the main obstacles to citizen mobility and, in the case of this study, on the obstacles to mobility using public transport.

For the proposed architecture to fulfill its objective, it is necessary to use different types of technologies, pertaining mainly to communications, computer systems and sensors. The systematic development and deployment of systems also entails the use of ITS architecture standards, with the aim of building systems that:

- Are easily integrated into existing technological infrastructure in transport networks.
- Have a high degree of interoperability so that they can operate in different public transport environments and, in addition, cooperate with other existing systems.
- Have a high degree of flexibility to enable expansion of their component parts, user base and locations in which they are implemented. This property implies scalable location: as intelligent spaces become more sophisticated, the interactions and number of devices and users will increase, affecting the communications bandwidth and energy consumption of the devices involved. For spaces located at heavily transited points of the transport network, this challenge becomes all the more relevant.

In addition, to enhance the usability of the systems developed using the proposed architecture, they must adapt autonomously to different environments in the existing transport network. To endow the model with an awareness of the different transport network contexts (context awareness), we applied the principles of ubiquitous computing in order to create systems able to build effective intelligent spaces [8]. An effective intelligent space is an open or closed area in the transport network in which ubiquitous applications, using communication infrastructures, distributed computing systems and sensors, provide accessible and useful information to the users of these spaces.

## 3. Related Studies 

This paper describes an architecture model for the systematic development of advanced information services in the context of public passenger transport by road. For the purposes of this paper, advanced information services are services that are able to process the context in which they operate and run autonomously. For this reason it is crucial, firstly, to use sensors that are deployed in transport infrastructure, especially those available in vehicles, and secondly, to use computing paradigms that are geared towards the development of intelligent environments in mobility contexts. The proposed architecture has therefore employed the models and techniques of ubiquitous computing. Finally, the systematic development of information services entails the development of user applications and processes based on the components, operating principles and design discipline that are provided by an architecture. The objective is for these services to have a high level of integration, scalability and interoperability. Therefore, the proposed architecture is based on the SOA model (service-oriented architecture).

In light of the preceding paragraph, this section on related studies first describes a range of relevant studies on ITS architecture models and the use of SOA in ITS development. As public transport plays a key role in transport management systems and this paper proposes an architecture model for public transport, we then address studies related to these systems and, in particular, those related to public transport, access to real-time information for route planning and systems designed to provide data to improve public transport and adapt it to the needs of its users. The last group of related studies illustrates the use of ubiquitous computing in developing traveler information services, the integration of different types of sensors, tracking systems and mobile communications technologies.

Since the 1970s, transport authorities have paid much attention to the field of ITS because it has developed systems that provide significant social and economic benefits. For example, in Europe, the aim of the Co-operative Networks for Intelligent Road Safety project (COOPERS) [9] and the Spanish project called “Operacion de Autopistas Seguras, Inteligentes y Sostenibles” (OASIS) [10] initiatives was to develop intelligent and cooperative systems to improve citizen safety and mobility. The result is a wide range of ITS cases that improve the efficiency, safety, accessibility and sustainability of transport systems. As a result of this large number of ITS cases and the technologies involved, the transport authorities of the most developed countries have promoted the development of recommendations and standards in ITS technologies and architecture. For example, Europe has an ITS architecture model called Extend Framework Architecture for Cooperative Systems (E-FRAME) [11], the USA has a model called National ITS Architecture [12], and Japan also has one [13]. Finally, ISO, the International Organization for Standardization, specifically its Technical Committee TC 204, has also developed standards for core ITS reference architectures: ISO 14813 [14].

In the context of ITS architecture models, there is a group of studies related to our proposal that use the SOA model to develop transport information systems. Use of this model enables distributed systems to be built with loose coupling between components. This paradigm is based on the use of metadata to describe web services, mechanisms for publishing these services used by information providers and mechanisms for discovering these services used by information consumer processes. The particularity of this model makes it suitable for achieving high levels of interoperability and technology independence in ITS [15]. Tao [16] used the SOA model to build an ITS to tackle the problem of traffic congestion in the city of Shanghai; Aloisio [17] described the potential of this model to integrate web applications and services in an ITS; and Dasheng [18] proposed an ITS architecture based on multi-agent SOA.

Transport Management Systems aim to improve the efficiency, reliability and safety of transport system operations. They provide reliable information that users may access from their homes, workplaces or places of recreation to plan their trips, or even during travel to obtain information on the route they are currently using (Diab [19]). This information is accessed using various types of mobile or fixed personal device and both fixed and mobile communications infrastructures. The provided information is georeferenced and relates to routes (planned or currently underway), timetables, fares, etc. An example of a system used by transport authorities in the ITS field is Automatic Vehicle Location (AVL) (Yan [20] and Padrón [21]). In a similar vein, Tran [22] proposed using the data from these systems to build service reliability diagnostic diagrams using Bayesian networks, in order to ascertain the variability of the attributes of the services and their effects on traveler behavior.

Public Transport Information Management Systems are a special type of Transport Management Systems that are based on the use of all types of data related to transport systems and their purpose is to generate knowledge to help understand past and present and predict the future of this type of transport. Therefore, they are a very useful tool for authorities and transport planners. In this context, big data and data mining techniques have particular relevance today, as they enable the processing of large volumes of data to obtain knowledge to interpret what is happening at each place and time on the transport network. Lathia [23] proposed algorithms to predict the time it takes to go from origin to destination stop, regardless of the route taken, based on the following patterns: first, the trip context, using data on travelers that make the same journey in one time interval; second, trip familiarity, which considers the frequency with which the traveler makes the journey; and third, a model that assumes that the traveler exhibits consistent habits when moving between the same two stops. Du [24], using the information recorded by automatic payment systems, estimated the behavior of citizens taking into account socio-demographic factors: location of shopping centers, sports facilities, residential areas, etc. In a study on the use of nearly 15,000 buses operating in Beijing, which carry around 17 million passengers daily, the objective was to ascertain the Group Mobility Pattern (GMP) in order to predict behavior in urban areas and to estimate the demand for public transport in areas of urban sprawl. Levner [25] proposed developing a system to predict and detect weak points on the transport network, based on data mining and multi-agent systems. This involves two types of analysis: offline processing, related to inadequate planning, and real-time processing to solve the problems arising from random events (accidents, unforeseen congestion). Zhou [26] conducted a study, based on information generated by vehicle GPS tracking devices, on how to avoid overcrowding, which, together with late arrivals, may deter citizens from using public transport services. Baloain [27] proposed a method for planning public transport networks in urban areas based on open and distributed collaboration in public information services (crowd sourced data), specifically using Waze and OpenStreetMap.

Traveler information systems are intended to provide traveler information irrespective of type (private or public) and mode of transport used (sea, air or land). Their areas of action may be regional, national or even international. These systems feed on data from systems that pertain to other ITS fields and, through different mobile communications infrastructures, provide information to travelers in private cars or public transport to enable them to plan or modify the next stages of their journey. Currently, they are a very important tool in government mobility policies, as they integrate all modes and types of transport (on foot, bicycle, private vehicle or public transport). Cases of this type of system include: the proposal of Barbeau [28] for transit riders with special needs, the proposal of Arikawa [29] for a pedestrian navigation system based on ubiquitous computing, the pedestrian navigation system based on context awareness and augmented reality proposed by Luna [30], and the proposal of Zhou [31] for a system that facilitates access to public transport for people with special needs based on ambient intelligence.

It is currently assumed that one of the ways to solve the problems caused by the mobility needs of people and goods is to develop intelligent transport systems capable of adapting to changing situations and needs in transport networks. These adaptive intelligent transport systems use a large number of data sources: sensors, in-vehicle systems, installed infrastructure systems, etc. These systems are also characterized by their ability to integrate different fixed and mobile communications infrastructures to process large amounts of data, making use of big data, data mining, multi-agent systems, etc. This is the context for our study, the main contribution of which will be to describe how to apply the principles of ITS architecture models to develop intelligent systems for public road transport. These intelligent systems handle a large—sometimes massive—amount of data provided by hardware and software components commonly deployed in the public transport network, especially in-vehicle sensors and other devices. Furthermore, these services are integrated into the infrastructure of the transport network without interfering with other systems that are running on it. The proposed architecture is therefore an intelligent management model for data communications that adapts to the communication capabilities that are already available on the transport network.

## 4. Description of the Architecture Model

**Figure 2:**
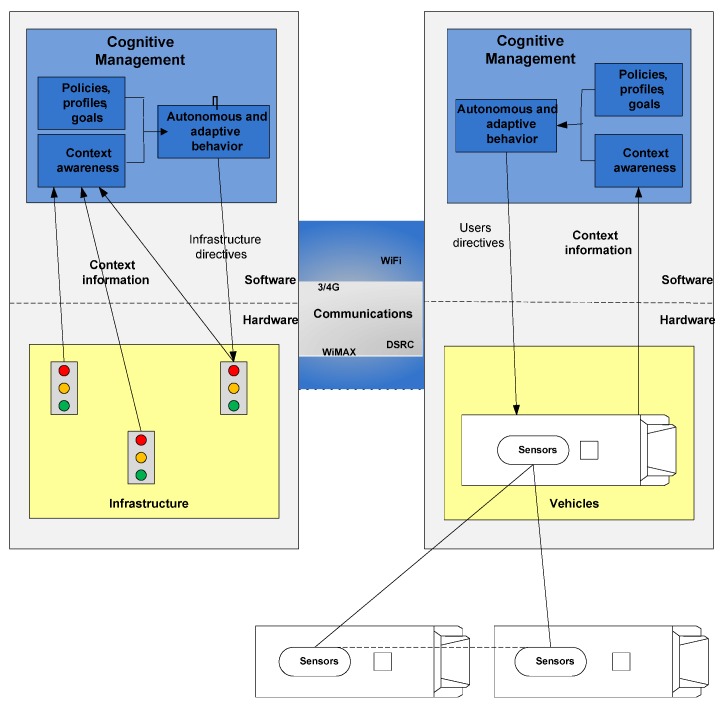
Concept illustration of the cognitive transport network based on the cooperation of all elements of the transport network (infrastructure, vehicles and users).

Figure 2 illustrates the transport system model that the proposed architecture will adopt. This model is based on technological advances in intelligent systems, communication technologies and sensors. Thus arose the concept of cognitive transport network as described by Dimitrakopoulos [32], in which the sensors located on infrastructure, vehicles and personal mobile devices obtain data, transmit them using different communication technologies (broadband or narrowband, long distance or local) and store and process them using advanced data processing techniques, such as big data or data mining, in order to obtain new knowledge to improve the public transport network.

**Figure 3:**
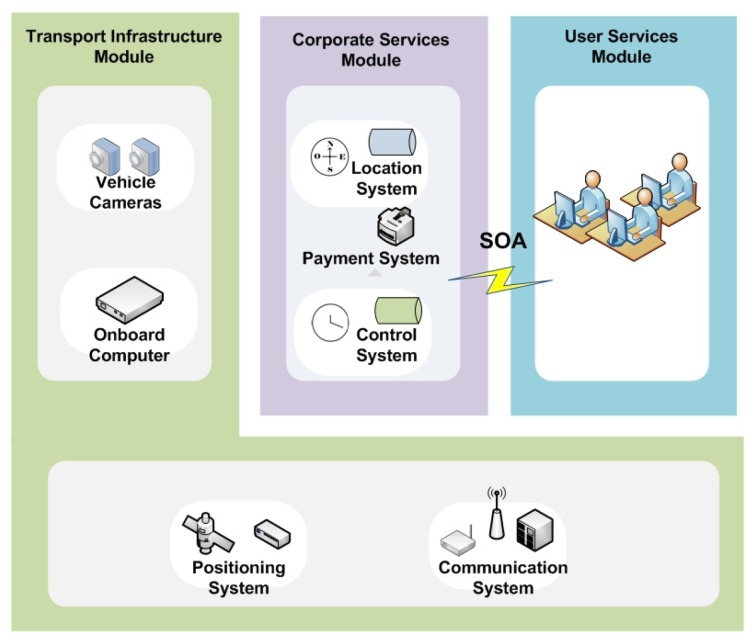
Overview of the proposed system architecture.

The proposed architecture model is based on ITS architecture recommendations, with the aim of ensuring the integration, interoperability and scalability of the services to be developed, and ubiquitous computing will ensure that these services are able to adapt autonomously to different environments and user needs. In addition, the proposed system model makes use of service-oriented architecture (SOA) in order to be able to use those infrastructure elements that are not standard, isolating their specificities from the other architectural elements. Figure 3 illustrates the proposed system model, which is divided into the three main modules described below.

### 4.1. Modules

#### 4.1.1. Transport Infrastructure Module

The Transport Infrastructure Module (TIM) comprises all the hardware and communications elements that make up the transport infrastructure. It consists of two subsystems: the communications subsystem and the subsystem of sensors and actuators that are deployed in the transport infrastructure.

The communications subsystem integrates all the communication technologies used in transport activity, enabling communication between vehicles and infrastructure (V2I, I2V), between vehicles (V2V) and between different infrastructures networks (I2I). They are basically composed of technologies and protocols including the following.

- Local communications: RS-232, RS-485, IR, Bluetooth (IEEE 802.15.1), ZigBee (IEEE 802.15.4), WiFi (IEEE 802.11), ITS G5 (IEEE 802.11p), etc.
- Long-distance communications: Fiber or copper networks, GSM, GPRS, 3G, 4G, WiMAX (IEEE 802.16), LTE, and iBurst.

The communications infrastructure is a horizontal component of the model, providing services to the remaining components of the model. In turn, it is organized in a layered structure composed of the following two modules.

- Transactions Module: Responsible for the required automatic flow between mobile and infrastructure systems being executed with a full guarantee of integrity, non-duplication and reliability.
- Network Module: Responsible for integrating and using different available communications technologies.

The sensors and actuators subsystem provides all the sensor technology that is deployed both on locations of the transport network (stations, stops, garages, etc.) and in vehicles, integrating:

- Infrastructure sensors, such as camera systems, ad-hoc wireless sensor networks (WSN), etc.
- Vehicle sensors, such as vehicular Ad Hoc Networks (VANETs) sensors for internal use in vehicles, etc.

#### 4.1.2. Corporate Services Module

The Corporate Services Module (CSM) comprises all the processes and services related to transport activity. Therefore, this module is the responsibility of transport operators and/or transport authorities. In turn, it comprises two subsystems: the infrastructure and transport activity monitoring subsystem and the services subsystem.

The infrastructure and transport activity monitoring subsystem provides all the data supplied by the infrastructure monitoring systems at stops and stations, and by the vehicle alarms and exceptions systems. Systems in the CSM module are classified into two groups:

- Acquisition: Responsible for acquiring any data that represent aspects that are relevant to production, both physical, such as the position or speed, and logical, such as vehicle status, customer demand, etc. These processes are linked to mobile platforms and devices available in transport infrastructure, such as cameras, sensors, information panels, etc.
- Monitoring: Responsible for verifying and quantifying the level of planning adjustments. They are able to detect events that may affect planning and transmit them for processing. These processes are executed on mobile platforms, and their design builds on network management and ITS architecture standards to achieve a high degree of scalability, interoperability and flexibility.

The services subsystem comprises the systems that provide data originating in the different activities related to transport. They can be classified into three groups.

- Control: The objective of these systems is to provide solutions to operating events that may occur, such as technical alarms, planning failures or unplanned events.
- Optimization: Consisting of systems that can adapt and thus improve the planning of operations to be carried out by vehicles.
- Information Provider: Comprising systems that present accessible information to system users. The main requirement that these processes must fulfill is to present the information regardless of the device used (mobile phone, driver console, fleet checkpoint, stop information panel, etc.).

To ensure that these services offer high availability at all times and places, this module integrates resources based on new computing paradigms such as cloud computing.

#### 4.1.3. User Services Module

The User Services Module (USM) deploys advanced user information services, such as route assistants, payment systems, emergency warning systems, guidance systems for people with special needs, etc. These services are built using the resources provided by the modules described above. Mobile user devices play an important role in accessing these services.

**Figure 4:**
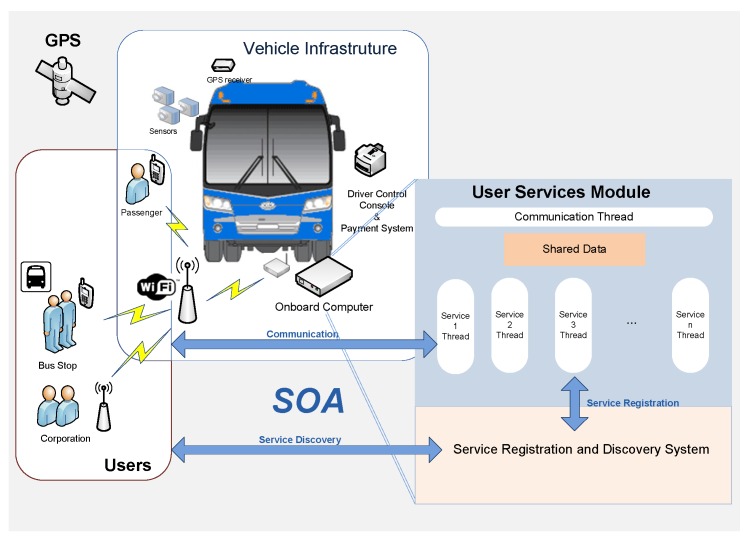
General Service operating schema.

The SOA model is used to build the interface between the CSM and USM modules. This model has been used to achieve a high degree of decoupling between the applications running on user devices and the systems pertaining to the CSM and USM modules. The central element of the SOA interface is the service registration and discovery system. Through the registration system, the data obtained by the sensors are accessed by the CSM services, which then advertise them so they can be discovered by the user applications. Figure 4 illustrates this general operating schema. Each service has a thread that runs on the onboard computer, and the data required for the service are transmitted by the different elements in the CSM using the communications system. When a service is ready to provide information, it registers and advertises its availability. Users connect to the infrastructure through their mobile devices and search for the required service. Every service has the following operating scheme:

- Step 1. Service start-up. Before providing data, all services must initialize their data and execution of the required resources.
- Step 2. Advertisement of service. Once start-up has been successfully completed, all services advertise their availability to potential consumers of their data.
- Step 3. Execution of service. At this point the service logic is executed, assuming that all required resources are available and initialized.

### 4.2. Context Awareness

In order for systems developed following the proposed model to behave autonomously, they must be able to identify the different contexts in which the transport vehicle operates. Having identified the different operational contexts, the systems may exchange data with each other and execute actions depending on the context. The proposed architecture provides a scheme that enables the systems to classify and therefore systematically identify the different contexts. This scheme uses two properties to classify the context: the first is the location of the vehicle and the second is the current operational status of the vehicle. The vehicle location property reports on where the vehicle is in the transport network; therefore, this information is provided by the vehicle location service. The values that have been identified as relevant are:

- Maintenance point (MP): At this location the vehicles are out of service due to maintenance or rest. Examples of this type of location are garages and workshops.
- Bus stop (BS): The vehicle is at a stop on the line it is servicing.
- Bus route (BR): The vehicle is traveling.

The operational status property indicates what the vehicle is doing. This information is provided by the vehicle operations control system. As is the case with the aforementioned property, the possible values to be considered for this property should be taken from data model standards. For our proposal, we have identified three relevant values:

- Out of service (O/S): The vehicle is not servicing a route and therefore cannot carry passengers.
- Line service (LS): The vehicle is servicing a route and carrying passengers between stops.
- Operational stop (OpS): The vehicle is in service but is not servicing a route.

To achieve interoperability in this context classification and identification schema, the proposed architecture adopts values or attributes based on conceptual data model standards, such as the Transmodel European standard [33]. All the contexts to be processed by the different systems may be classified by a pair of values. Table 1 is a matrix in which each of the elements represents a different context and actions that are being performed are specified in each possible context. The rows correspond to the vehicle operational status (O/S, LS and OpS) and the columns indicate the location on the transport network (MP, BS and BR).

### 4.3. Intelligent Data Management

In our proposal, data management is a key element since, in transport systems and especially in large-scale transit systems, information systems must handle large volumes of data that are occasionally provided by mobile systems, such as onboard vehicle systems and user devices. To address this challenge, our proposal is based on the ubiquitous systems data management model, which, according to Perich [34], has the following main features:

- Autonomous: They have a high degree of autonomy given the lack of centralized control of the data handled by the ubiquitous applications.
- Distributed: The data being handled are distributed, which means that they are structured in different devices, and there are various copies of the data.
- Heterogeneous: The data represent heterogeneous ontologies.
- Mobile: Any application on a ubiquitous system is intrinsically mobile, since the system running the application changes its location, so communication infrastructures are also changing, which is why the same set of data is not always available in the system.

Consequently, ubiquitous applications, when taking data management into account, are characterized by their ability to:

- Operate in environments where the number of data applications and spaces is dynamic.
- Operate with different data catalogues and schemas.
- Operate without any guarantee of reconnection despite the high risk of creating inconsistencies in the data.
- Provide a platform for controlled collaboration.

**Figure 5:**
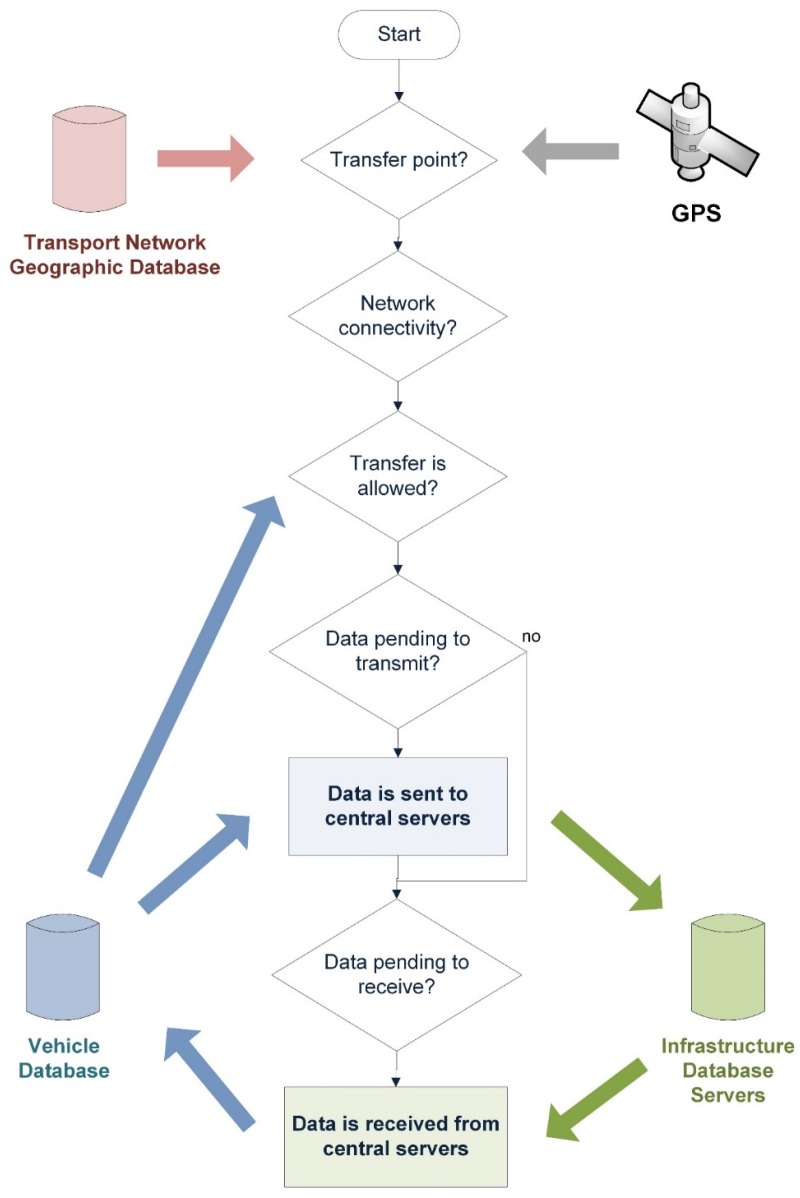
Schema of autonomous data transfer between vehicles and infrastructure.

The architecture is aimed at providing advanced information services in the context of the public transport of passengers by road. Examples of this type of service are public transport route assistants or the monitoring of operations being carried out by each vehicle of a public transport fleet. Both examples of systems are characterized by the fact that they handle a large amount of data, since they need a representation of the transport network, i.e., all vehicle stops and stations including geolocation data. Moreover, data representing the different routes covered by the vehicles are required, as is specific information on their operations schedule along various points of the transport network. Ultimately, the transfer of large amounts of data from mobile systems to infrastructure data repositories is necessary to synchronize the different databases. With the classification and recognition of the different contexts, the architecture provides a framework for these data transfers to be performed autonomously in such a way that guarantees their integrity. Figure 5 illustrates this schema, in which data transfers are performed using a local mobile communication technology, such as WiFi, and are executed when the vehicle is at a point of the transport network where there is communication coverage. In our proposed model, these points are referred to as Transfer Points, and are places on the transport network where vehicles are stationary for significantly long scheduled intervals, such as garages, stations or line stops. In addition, transmission takes place only when the vehicle is moving at a speed below a threshold that depends on the communications technology being used. For example, in the application of this architecture to the real case of a transport company this threshold was set at 10 km/h, due to, firstly, the fact that the company’s WiFi infrastructure operates in the 2.5 GHz band and, secondly, the range of the access points. It was found that when vehicles circulated at speeds above this threshold a considerable number of data transfer integrity errors occurred. Owing to both the limitations of the technology currently employed (it only operates in the 2.4 GHz band) and the range of the access points, the time required for transfer above 10 km/h is insufficient in most cases to complete the transfer with the appropriate guarantees of integrity at origin and destination. The point at which the vehicle is located is provided by the vehicle location system, speed, GPS receiver, the time it will remain stationary at that point, and the vehicle’s operational monitoring system.

### 4.4. Architecture Assessment

The proposed architecture model was used to develop various services for the information system of the transport company Global Salcai-Utinsa S.A. This public passenger transport company operates on the island of Gran Canaria, in the Canary Islands (Spain), covering the island’s interurban transport. It currently has a fleet of 304 buses, covering 28,897,002 km and transporting 19,284,378 passengers a year. Two of the services developed using the principles and elements of the proposed architecture are the production control system, which monitors all vehicle activity, and the planning control system, which monitors vehicle operations according to the company’s operations schedule. Both are examples of services that handle a lot of data, because they require vehicles to have a database that includes a geographical representation of the transport network, detailed planning of vehicle operations, and they must register each relevant event that occurs in each vehicle. This wealth of data is managed intelligently by the communications system of the proposed architecture.

The architecture was assessed from two different points of view. The first assessment analyzed to what extent the design goals had been achieved for each of the services developed, using a set of qualitative indicators. The second assessment obtained values that show how the services based on the proposed architecture perform. Considering the importance of the communications system in the architecture, there are some communications system performance parameters that affect all the services, affecting them more as the volume of data required by the services increases.

#### 4.4.1. Qualitative Indicators of the Architecture

These indicators verify to what extent the design goals of the proposed architecture have been achieved. Based on the design goals described in Section 2, the following qualitative indicators were established:

- Ease of service integration: The indicator to evaluate this design goal is the number of hardware/software components required to run the service on the infrastructure.
- Interoperability: To assess the capability of the service to run in different configurations of the infrastructure elements and its capability to cooperate with other key services of the hardware and software components used to connect the service to the infrastructure.
- Flexibility: To assess the expandability of the service, adding new hardware/software components to the configuration or increasing the number of users.
- Fault tolerance of the systems in the event of failure of infrastructure components: This is to verify whether the service provided by a system can continue to be available if a component of the required infrastructure fails. To assess this capability it is necessary to analyze whether the failure of a component required by the service causes its unavailability, or if, on the contrary, it continues to run because the component that failed has been replaced automatically by another.

#### 4.4.2. Architecture Performance Analysis

As mentioned above, the communications system significantly affects the way the services perform, especially if they require the handling of a considerable amount of data. For this reason, the performance parameters of the communications system were used as common performance indicators for the systems that we developed. The parameters used for assessing performance were:

- Ability to handle large volumes of data: As already indicated, in order to tackle major mobility problems, ITS must now handle a lot of data from different sources: sensors, user devices, embedded systems, etc. Therefore, a first performance criterion is verifying the ability of the architecture to handle these large volumes of data.
- Error rate in data transfers, especially between vehicle and infrastructure: This parameter is the percentage of erroneous transmissions of data files produced by the communications system. There are two factors that affect this error rate. The first is related to failures in the communications infrastructure (Network Module); the second, to spontaneous connections/disconnections of the systems due to their mobility (Transactions Module). In the case of the vehicles, and following the schema of autonomous transmission described in Figure 5, the more predictable the vehicle behavior, i.e., the extent to which it complies with planning, the less the spontaneity of the connections/disconnections will affect the data transfer error rate between vehicle and infrastructure.
- Latency in data transfers: This is the time from when the connection starts to transmit a data file until it arrives, free of errors, at its destination, which may be a vehicle component, an infrastructure system or a user device. In the case of data transfers between vehicle and infrastructure, this time depends not only on the bandwidth provided by the architecture’s network module, but also on the amount of data to be transmitted. This is because, depending on this amount of data to be transmitted, the autonomous transmission of data incorporated in the architecture (see Figure 5) decides when to transmit based on the vehicle’s position, speed, the status of the vehicle according to planning, and the time that it will remain in that status.
- Response time in the interactions between the user and the service’s user application: This is the time required by the user application to notify the user of any relevant service event. It is a performance parameter appropriate for systems requiring user interaction.

**Figure 6:**
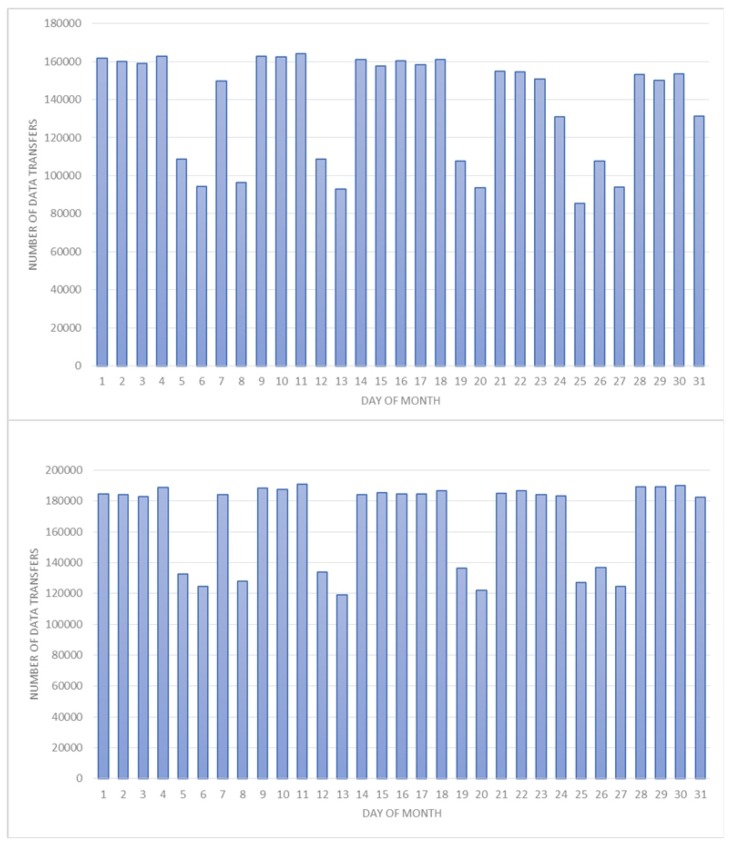
Number of data files transferred between the vehicles of the fleet and the corporate data repositories for the month of December 2015: (**Top**) the files of the production control system; and (**Bottom**) the files of the planning control system.

The first three performance parameters—amount of data transferred, error rate and latency—are parameters that can be used as a measure of performance for any service developed in the architecture, since any service must exchange data with the corporate data repositories. However, these parameters are especially relevant for those services that need to handle large volumes of data, such as the geographical data of the transport network, data related to payment systems supported on vehicles, planning data, data generated during vehicle activity or even updating a program running on the onboard computer. Figure 6 shows the data transmitted by the communications system during the month of December 2015. These data are generated in the vehicle by the production control system and the planning control system, and each column represents the number of files transferred daily. The top graph shows the files transferred between the vehicles and the corporate data repository of the production control system; the total number of files transferred by this system was 4,250,952, which, expressed in megabytes, and considering the average size of the different file types, is 101.35 megabytes. The bottom graph shows the files transferred between the vehicles and the corporate data repository of the planning control system; the total number of files transferred was 5,191,619, which, expressed in megabytes, and considering the average size of the different file types, is 99.02 megabytes. The error rate in these data transmissions was 10%.

## 5. Use Case: Bus Stop Network Monitoring System in Geographically Remote Areas

In public transport networks, especially in mass transit systems, security against vandalism or criminal acts is a requirement for the transport authorities. For this reason, surveillance systems based on image and sound sensors are commonly used in sensitive areas of transport infrastructure, such as stations, bus stops, public transport vehicles, etc. The signals produced by the sensors of these systems are transmitted using dedicated communications systems. When the transport network has to cover large geographic areas, including remote rural areas, monitoring the infrastructure deployed in these places is often a technological challenge because these areas sometimes lack basic elements such as electricity and communications. This situation occurs frequently in the case of public passenger transport by road that needs to cover remote rural areas. This section describes how we applied the proposed architecture model in the development of a bus stop monitoring system. Using this Bus Stop Monitoring System (BMS), transport authorities and operators can monitor the status of the stops, i.e., fittings, condition, elements that potentially hinder accessibility, etc.

**Figure 7:**
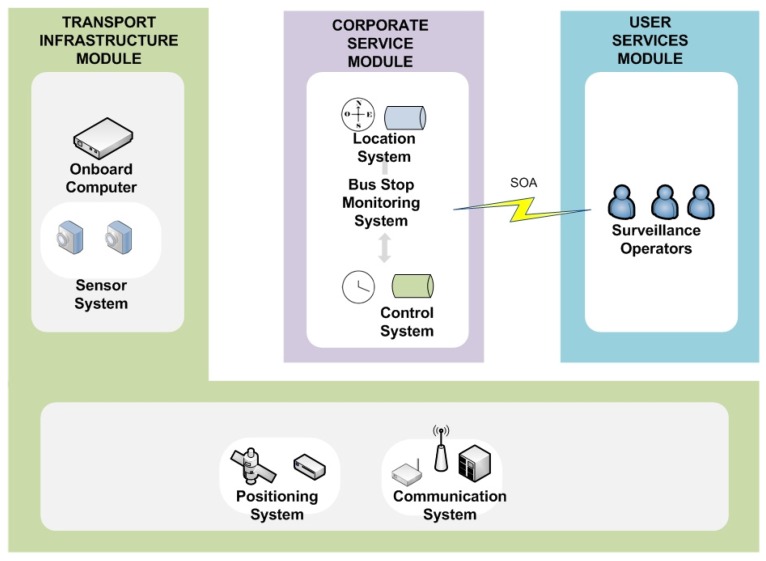
Overview of the bus stop monitoring system.

To overcome the lack of basic resources in remote places, BMS uses the sensor, computing and communications resources on the public transport vehicles that constantly service these locations. The specific vehicle resources are: two image sensors to acquire images outside the vehicle; the location system to detect when the vehicle is at a stop to be monitored; the onboard computer running processes to compress and store the acquired images as well as processes that endow the system with intelligent behavior; and a communications system for the transfer of data and images. System operation is based on selective processing in different contexts of the transport network, these contexts being specific locations on the transport network: stations, stops and bulk data transfer points. Figure 7 gives an overview of the BMS that results from applying the proposed architecture, showing the three modules of the architecture: TIM, CSM and USM, and the elements used in each.

At the TIM level, the two sensors used have 23 LEDs that provide infrared light with a range of 20 meters. Due to the environmental conditions in which they have to operate, these sensors meet the IP66 specifications (protection against dirt and water), have high sensitivity in low light conditions and are equipped with automatic control of gain, brightness, contrast and viewing mode (day and night). Intelligent control of the image sensors is carried out using the onboard computer, which is equipped with a low-power processor, 2 GB of main memory, a solid-state disk of 64 GB, a network interface and four serial communication interfaces. Also at this level, serial communications are used on the network subsystem to control the image sensors. A WiFi infrastructure is used to transfer images between the vehicles and the central image repository. Finally, for emergency communications and alarms 3G technology is used.

In the CSM module, the corporate services that provide information to the system are the vehicle location system, which enables the vehicle to be located in the transport network, and the operations control system, which provides information on the operation being performed by the vehicle according to the established operations plan. With this information, the monitoring system knows at all times in which context of the transport network the vehicle finds itself and can intelligently control sensors and communications.

At the user services level, USM, the operators responsible for monitoring the condition of the transport infrastructure are connected via web services to image repositories to check the status of the stops, and can even connect to image repositories located in vehicles.

### 5.1. Operating Principles

**Figure 8:**
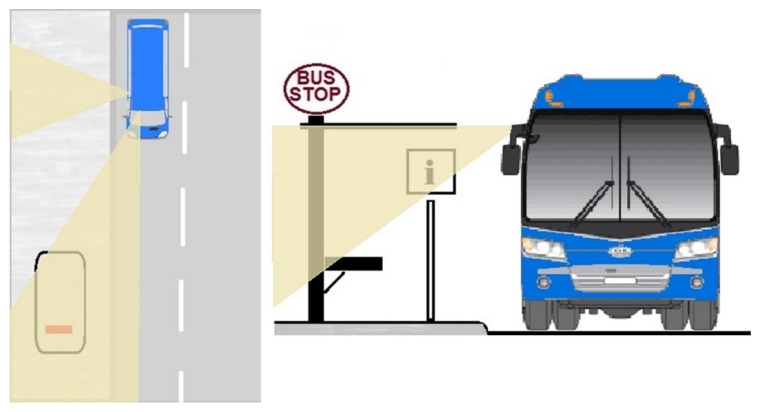
External vehicle sensors.

As has already been mentioned, the system has two image sensors for acquiring images outside the vehicle (Figure 8). One captures a panoramic front view of the road to detect elements that may potentially impede parking at the bus stop; the other is located on the right side of the vehicle and its purpose is to obtain images that enable the condition of the stops to be monitored.

**Figure 9:**
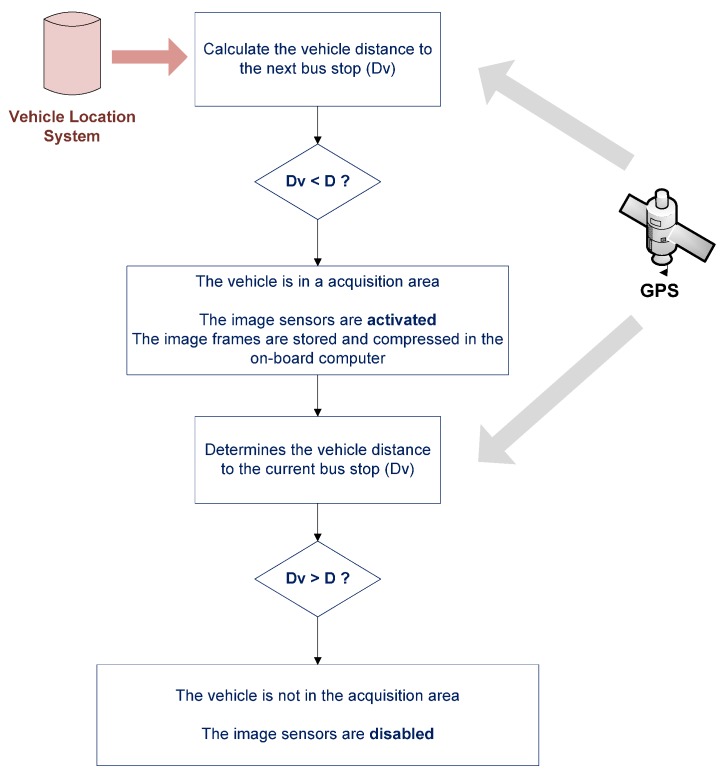
Intelligent control of image sensors.

When the vehicle begins service, the BSM is informed by the TIM module and the CSM module of the status of the required resources. The TIM module reports on the status of the image sensors and communications system, indicating whether they are operational or whether any of them are not. The CSM module reports on whether the location and operations control services are operational. If all the required resources are available, the BMS registers as an available service on the vehicle. Through this service registration, other systems on the vehicle or even at the central office, in an emergency and using the 3G communications system, can access the images acquired by the BSM. The BSM registry is associated with the “vehicle in service” context. This context is identified by the operations control system. When the vehicle approaches a stop, the external vehicle sensors are activated, and will remain active while the vehicle is within a radius proximity (D) of the stop; this is a variable parameter of the system, expressed in meters, which may be established according to various factors such as the route or features of the stop. This selective control of the external image sensors associated with the “vehicle at stop” context is performed by the vehicle tracking system. Using the vehicle position provided by the GPS receiver and the geographic database stored on the onboard computer, this system is capable of identifying at what point of the transport network the vehicle is at all times. The external sensors are activated and deactivated by calculating the distance between the vehicle position and the next stop on the route. When this distance is less than D, the sensors will be active and acquiring images. When the distance is greater than D the sensors are disabled. The distance used is the Euclidean distance, and is calculated by taking the following coordinates: the latitude, longitude and height of the stop and the position of the vehicle (see Figure 9).

**Figure 10:**
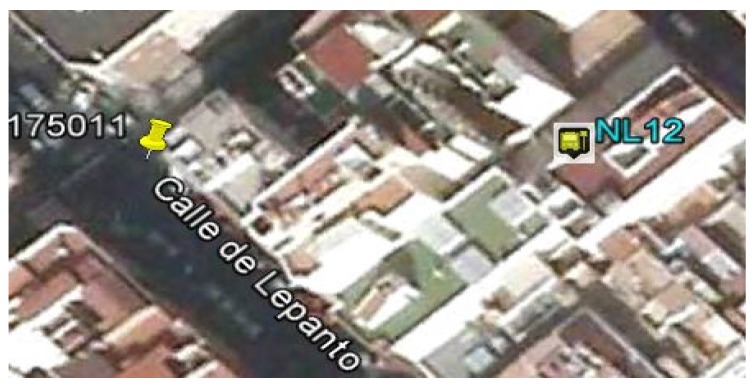
Aerial view of an example of GPS error at a stop in an urban environment.

The value of the parameter D must be set so that the acquisition area includes the entire stop. This requires the image sensors to be activated before reaching the stop and deactivated after passing it by. To achieve this behavior in the control of the sensors, the following factors have been taken into account:

- GPS measurement error is an error that may be due to different factors (ionosphere, troposphere, multipath, receiver noise, orbit errors, satellite clocks), which affect it differently. Currently, there are sophisticated GPS systems for civilian applications that incorporate mechanisms to mitigate some of these errors, but in our case the vehicles use a standard GPS receiver that does not correct such errors. In the studies that we have carried out, most of the errors occur in urban areas, where buildings impede reception of the GPS signal, causing errors of up to 50 m at times (multipath error). Figure 10 illustrates an example of this type of error; it shows the correct position of the stop (represented by icon 175011) obtained by a process of statistical sampling and the position of a set of GPS measurements with an error of around 50 m (represented by icon NL12).
- Another factor we considered when setting this value is that, in the geographical representation used by the transport company for which we developed the system, stops are represented by the geographic position of a point located at one end of the stop and the length of the stop varies, depending on the type of stop. In the case of the company for which the system was tested, the length of the stops can vary between 15 and 50 m.
- Finally, the system uses Euclidean distance as its distance function, which entails incurring minimal error, since this magnitude measures the distance between two points on a plane and not on a curved surface.

Therefore, parameter D adopts a variable value dependent on the characteristics of the stops. Considering these factors, the value of parameter D varies depending on the length of the stop: it may vary from 100 m, for the stops of minimum length, to 200 m, for the stops of maximum length.

**Figure 11:**
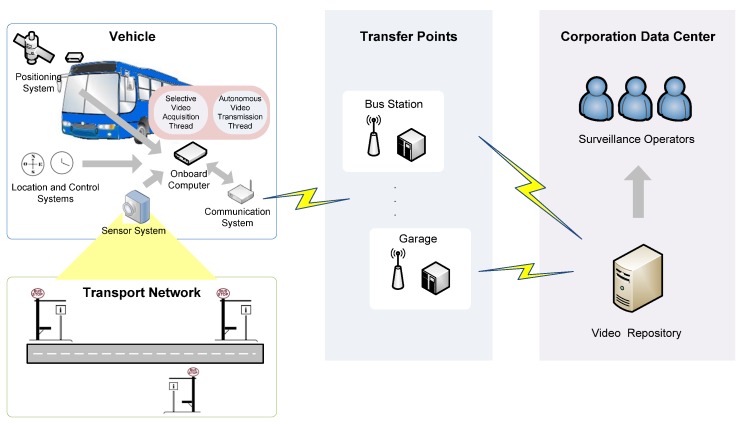
Surveillance system operation and elements.

With regard to communications, they are executed from vehicle to infrastructure (V2I), to transfer data and images stored on the vehicle to the central repository of the transport operator or authority, and from infrastructure to vehicle (I2V), to update the data used by the system, for example the operational plan of the vehicle or the geographic database of the transport network. Figure 11 illustrates how the system operates and its component elements.

Using the context representation matrix in Table 1, the system operating principles associated with different contexts are represented in Table 2.

### 5.2. System Assessment

This section will assess the use case that has been presented. The analysis criteria described in Section 4.4 were used to conduct the assessment. 

#### 5.2.1. Qualitative Analysis

As already indicated, the purpose of this analysis is to determine to what extent the design goals have been achieved, using the following indicators: ease of integration, interoperability, flexibility and service availability.

With regard to ease of integration, the system presented as our use case was integrated by installing cameras in the vehicle and connecting them to the onboard computer via a video-capture device. Because the software architecture is thread-based, the software is integrated into the systems with ease. The system software has a multi-thread structure formed by the service thread responsible for the service that controls the installed cameras. The thread responsible for obtaining all data required by the various services is run on the onboard computer via the communications system. These data are stored in a shared memory area that is accessible to all service threads running on the computer. Therefore, the service was integrated by incorporating only the hardware/software components of the system, which in this case are the cameras, the video-capture device, and the service software module, without changing the configuration of existing infrastructure elements.

With regard to interoperability, the new system obtains data provided by other components of the onboard system—vehicle speed from the positioning system, the location from the tracking system and the current operation to be performed from the planning control system—in a transparent way, accessing the shared memory area without using new communication elements and integrating it as described in the previous point.

Expansion of the system may be motivated by the need to use more cameras in vehicles, or to add more data transfer points to the network. The first of these needs would be satisfied using the ports available on the video-capture device and adding a new thread to control each camera to be added to the configuration. The second need would be satisfied by installing the necessary network infrastructure for data transfers, which would basically consist of WiFi access points and network switches.

With regard to the fault tolerance of the system, there are two critical aspects: image acquisition on the network of stops and transfer of these images. In a vehicle, the system may fail due to a malfunction of the onboard computer, the positioning system, tracking system, cameras or communications system. However, the other vehicles would provide images that they have acquired thereby ensuring monitoring of the stops through images from the network. Furthermore, the level of availability of the communications system for image transfers would increase by using a redundant infrastructure for WiFi communications at the transfer points and increasing the number of transfer points, especially at points of the transport network where vehicles are concentrated: stations and garages.

#### 5.2.2. Performance Analysis

**Figure 12:**
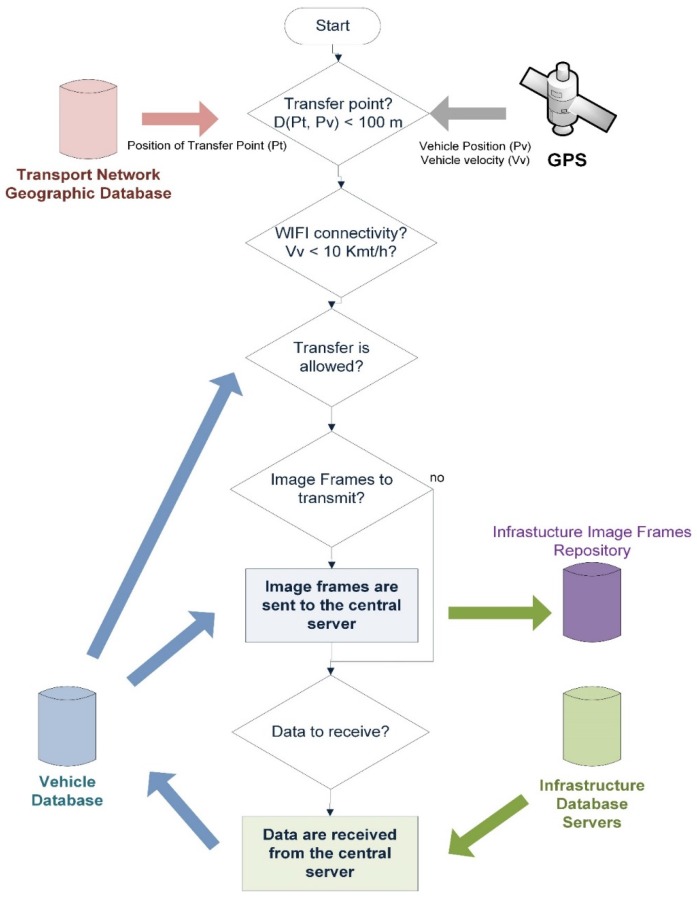
Flow diagram illustrating data transfer from vehicles to corporate repositories.

As we have already indicated, a large amount of data is involved in the BMS, including image files, a geographic database of the transport network and the operations plan. Therefore, one requirement of the system is intelligent management of the communications infrastructure, so that it can be run using the WiFi infrastructure. We have already mentioned that this technology has some limitations, one of which is that the transmission error rate is high when the mobile station is moving at speeds above 10 km/h. Another limitation to bear in mind is that the bandwidth varies depending on the distance between the mobile system and the WiFi access point and the obstacles that might hinder transmission. For our proposed system, the bulk data transmission scheme, described in Section 4.4 and illustrated in Figure 5, has been set up as illustrated in Figure 12. In this setup of the transmission scheme, data transfer begins when the vehicle is less than 100 m from the transfer point, is traveling at a speed below 10 km/h and will be in that area for long enough to complete the transmission.

To study the performance of the communications system, we analyzed the time required to transmit data from the vehicles to the central repository and the transmission error rate. Of all the data types handled by the system, the biggest files are the frames. It is therefore necessary to specify certain aspects of the images that are processed by the system. First, the resolution used in the tests was 640 × 480 pixels; second, the images acquired by the cameras have been compressed, so take up less storage space and less bandwidth when they are transferred. The method used by the compression process running on the onboard computer is H.260. With these specifications a one-second sequence of images requires on average 34.13 KB for storage. When analyzing the storage requirements and transmission time required for monitoring the different stops on the routes run by the vehicles, it should be borne in mind that the total time that the two image sensors are active at stops is composed of three periods:

- Approach time (T0): The time it takes the vehicle to travel the 100 m prior to reaching the stop. This time depends on the approach speed of the vehicle, which in turn depends on variables such as traffic conditions and road signs.
- Dwell time (T1): The length of time the vehicle spends at the stop for travelers to board or alight. This time depends on the number of passengers using the bus stop.
- Clearance time (T2): The period of time between the vehicle setting off from the stop and covering the following 100 m of the route. As with T1, this time depends on the speed of the vehicle, which in turn depends on variables such as traffic conditions and road signs.

Due to these factors, it is not possible a priori to predict the system’s storage space, bandwidth and data transfer time requirements. We have estimated these parameters based on the average data requirements on a representative route. The selected route runs through urban areas with high traffic density and remote rural areas with little traffic. We analyzed the behavior at 20 stops with different levels of passenger use. Table 3 shows the results for the route that we studied. It begins with an urban section, from stops 1 to 10, then continues through a stretch where the stops are isolated, from stops 11 to 17, and ends with another urban section, from stops 18 to 20. The second column shows the average time that the test vehicle is less than 100 m away from the stop. The third column contains estimates of the space required to store the images obtained by the two sensors at each stop during the average time noted in the second column. Finally, the fourth and fifth columns show the estimated time required to transmit these images depending on the distance from the vehicle to the WiFi access point. In the last row the totals for each column are given; this is the average total acquisition time for all stops, the average space required to store the images acquired by the two sensors at every stop during this average period of time and, finally, the average time required to transmit the images.

With regard to transmission errors, these occur mainly for two reasons. The first is that the WiFi network disconnects from the mobile system during the data transfer process. The second reason is that sometimes the vehicle GPS system supplies erroneous location data, causing data transfer between the vehicle and the infrastructure to start when the vehicle is traveling at speeds above 10 km/h. Each data file exchanged between the vehicle and the infrastructure creates a logfile in the system, notifying whether the transfer was successful or an error occurred. In the event of an error, the system retries until transfer has been successfully completed. If the vehicle is disconnected from the infrastructure during data transfer, the system will try to send the unsent files during the next connection. Analyzing these logfiles we have observed a file transmission error rate of around 7%.

## 6. Conclusions

We have presented an architecture model for the development of intelligent information systems for public road passenger transport. The model is based on international ITS architecture model standards with a view to providing a framework for the systematic development of these systems, endowing them with desirable properties such as interoperability, flexibility and ease of integration. In order to equip the systems with the capacity to automatically recognize the different environments of the road transport network and enable the systems developed using this architecture model to work intelligently, the proposed architecture has adopted the ideas and techniques of ubiquitous computing. In our proposed architecture, this intelligent behavior is used to provide an intelligent data management scheme that enables transmission of large amounts of data using mobile communication technology with limited reach and bandwidth. It has also used the service-oriented distributed system design model, the SOA model, which gives systems based on this architecture a high degree of integration with other systems already deployed in the infrastructure.

As a use case of the proposed architecture, we have described a monitoring system for bus stops on a road transport network. This system uses the equipment on the vehicles—image sensors, computer and communications systems—to acquire images of the stops and transmit them to the control centers so that authorities and operators may know the state of the equipment at those stops and detect accessibility problems. Operation of the system is based on selective processing in different contexts of the transport network, i.e., specific locations: stations, stops and bulk data transfer points. Thus, intelligent control of image sensors and communications is achieved. This system is specially designed to monitor parts of the transport network that are located in remote areas, and that therefore lack basic resources such as electricity and communications infrastructure.

We have identified several future lines of research. The first is to develop new intelligent systems using the proposed model. A specific objective is to develop a system to record and communicate, in real time, different aspects of the vehicle and its surroundings, such as speed and the weather and environmental conditions of the locations it travels through. In this way the vehicle becomes an active element in the provision of data that can be used to develop advanced information services for citizens in the context of smart cities or metropolitan areas. Another line of future research involves the development or improvement of the services that we have developed, such as the monitoring system, using mobile communication technologies with wide bandwidth and long reach, such as WiMAX technology.

## Figures and Tables

**Table 1:** Classification matrix of the different possible contexts and examples of actions to be taken in each.

	MP	BS	BR
**O/S**	V2I communications. I2V communications.	Context not covered.	Context not covered.
**LS**	Context not covered.	Vehicle status registration. Service registration. Service discovery. Provision of en route services.	Vehicle status registration. Provision of en route services.
**OpS**	Context not covered.	V2I communications. I2V communications.	Vehicle status registration.

**Table 2:** Bus stop surveillance system context matrix.

	MP	BS	BR
**O/S**	Image and data transfer from vehicle to infrastructure (V2I communications). Data transfer from infrastructure to vehicle (I2V communications).	Context not covered.	Context not covered.
**LS**	Context not covered.	Image acquisition at stops.	Vehicle status registration. Compression processes for acquired images.
**OpS**	Context not covered.	Image and data transfer from vehicle to infrastructure (V2I communications). Data transfer from infrastructure to vehicle (I2V communications).	Vehicle status registration. Compression processes for acquired images.

**Table 3:** Results obtained for representative route.

Stop	Average T. (s)	Estimated Average Required Storage Space (KB)	54 Mbits/s 75 m (s)	24 Mbits/s 140 m (s)
1	12.32	840.96	0.12	0.27
2	17.06	1164.52	0.17	0.38
3	16.30	1112.64	0.16	0.36
4	15.75	1075.10	0.16	0.35
5	16.93	1155.64	0.17	0.38
6	15.42	1052.57	0.15	0.34
7	16.97	1158.37	0.17	0.38
8	16.64	1135.85	0.16	0.37
9	17.19	1173.39	0.17	0.38
10	14.87	1015.03	0.15	0.33
11	15.56	1062.13	0.15	0.35
12	15.35	1047.79	0.15	0.34
13	15.16	1034.82	0.15	0.34
14	15.82	1079.87	0.16	0.35
15	15.96	1089.43	0.16	0.35
16	17.86	1219.12	0.18	0.40
17	15.05	1027.31	0.15	0.33
18	15.85	1081.92	0.16	0.35
19	16.21	1106.49	0.16	0.36
20	10.40	709.90	0.10	0.23
TOTAL	312.67	21342.85	3.09	6.95

## References

[1] Zhang J., Wang F., Wang K., Lin W., Xu X., Chen C. (2011). Data-driven intelligent transportation systems: A survey. IEEE Trans. Intell. Transp. Syst..

[2] Shawe-Taylor J., de Bie T., Cristianini N. Data mining, data fusion and information management. Proceedings of the 2006 IEEE Intelligent Transportation Systems Conference.

[3] Peters A., von Klot S., Heier M., Trentinaglia I., Hörmann A., Wichmann H.E., Löwel H. (2004). Exposure to traffic and the onset of myocardial infarction. N. Engl. J. Med..

[4] European Commission. http://europa.eu/rapid/press-release_IP-13-236_es.htm.

[5] Malta L., Miyajima C., Takeda K. (2009). A study of driver behavior under potential threats in vehicle traffic. IEEE Trans. Intell. Transp. Syst..

[6] Federal Highway Administration. http://ntl.bts.gov/lib/jpodocs/rept_mis/7243.pdf.

[7] Sussman J.M. (2005). Perspectives on Intelligent Transportation Systems (ITS).

[8] Satyanarayanan M. (2001). Pervasive computing: Vision and challenges. IEEE Pers. Commun..

[9] European Commission COOPERS. http://cordis.europa.eu/project/rcn/79301_en.html.

[10] INDRA. http://www.indracompany.com/en/indra/oasis-operacion-autopistas-seguras-inteligentes-sostenibles.

[11] European Commission’s Directorate General for Mobility and Transport. http://www.transport-research.info/web/projects/project_details.cfm?id=44407.

[12] United States Department of Transportation. http://www.iteris.com/itsarch/index.htm.

[13] Comprehensive Plan for ITS in Japan. http://www.mlit.go.jp/road/ITS/5Ministries/index.html.

[14] International Standards Organization (ISO). http://www.iso.org/iso/catalogue_detail.htm?csnumber=43664.

[15] International Standards Organization (ISO). http://www.iso.org/iso/iso_catalogue/catalogue_tc/catalogue_detail.htm?csnumber=42014.

[16] Tao X., Jiang C., Han Y. Applying SOA to intelligent transportation systems. Proceedings of the 2005 IEEE International Conference on Services Computing.

[17] Aloisio G., Carteni G., Sponziello A., Laudadio T. Design strategies for web based ITS applications—A proposed architecture in design of intelligent transport systems application. Proceedings of the International Joint Conference on e-Business and Telecommunications.

[18] Wang D., Ren L., Li J. Modeling intelligent transportation systems with multi-agent on SOA. Proceedings of the 2010 International Conference on Intelligent Computing and Integrated Systems (ICISS).

[19] Diab E.I., El-Geneidy A.M. (2012). Understanding the impacts of a combination of service improvement strategies on bus running time and passenger’s perception. Transp. Res. Part A: Policy Pract..

[20] Yan Y. (2012). Bus transit travel time reliability evaluation based on automatic vehicle location data. J. Southeast Univ..

[21] Padrón G., García C.R., Quesada-Arencibia A., Alayón F., Pérez R. (2014). Using massive vehicle positioning data to improve control and planning of public road transport. Sensors.

[22] Tran V.T., Eklund P., Cook C. (2014). Learning diagnostic diagrams in transport-based data-collection systems. Found. Intell. Syst..

[23] Lathia N., Smith C., Froehlich J., Capra L. (2013). Individuals among commuters: Building personalized transport information services from fare collection systems. Perv. Mob. Comput..

[24] Du B., Yang Y., Lv W. Understand group travel behaviors in an urban area using mobility pattern mining. Proceedings of the IEEE 10th International Conference on Ubiquitous Intelligence and Computing.

[25] Levner E., Ceder A., Elalouf A., Hadas Y., Shabtay D. Detection and improvement of deficiencies and failures in public transportation networks using agent-enhanced distribution data mining. Proceedings of the IEEE International Conference on Industrial Engineering and Engineering Management.

[26] Zhou C., Dai P., Li R. The passenger demand prediction model on bus networks. Proceedings of the IEEE 13th International Conference on Data Mining Workshops—ICDMW 2013.

[27] Baloian N., Frez J., Pino J., Zurita A.G. Efficient planning of urban public transportation networks. Proceedings of the 9th International Conference on Ubiquitous Computing and Ambient Intelligence—UCAmI 2015.

[28] Barbeau S.J., Winter P.L., Georggi N.L., Labrador M.A. (2010). Travel assistance device: Utilizing global positioning system-enabled mobile phones to aid transit riders with special needs. IET Intell. Trans. Syst..

[29] Arikawa M., Konomi S., Ohnishi K. (2007). Navitame: Supporting pedestrian navigation in the real world. IEEE Perv. Comput. Mob. Ubiquitous Syst..

[30] Luna J.M., Hervás R., Fontecha J., Bravo J. A friendly navigation-system based on points of interest, augmented reality and context-awareness. Proceedings of the 6th International Conference on Ubiquitous Computing and Ambient Intelligence—UCAmI 2012.

[31] Zhou H., Hou K.-M., Zuo D., Li J. (2012). Intelligent urban public transportation for accessibility dedicated to people with disabilities. Sensors.

[32] Dimitrakopoulos G., Demestichas P. (2010). Intelligent transportation systems based on cognitive networking principles and management functionality. IEEE Veh. Technol. Mag..

[33] Transmodel: Reference Data Model for Public Transport. http://transmodel-cen.eu/.

[34] Perich F., Joshi A., Finin T., Yesha Y. (2016). On data management in pervasive computing environments. IEEE Trans. Knowl. Data Eng..

